# Radiation-Induced Reprogramming of Pre-Senescent Mammary Epithelial Cells Enriches Putative CD44^+^/CD24^−/low^ Stem Cell Phenotype

**DOI:** 10.3389/fonc.2016.00138

**Published:** 2016-06-14

**Authors:** Xuefeng Gao, Brock J. Sishc, Christopher B. Nelson, Philip Hahnfeldt, Susan M. Bailey, Lynn Hlatky

**Affiliations:** ^1^Inserm UMR 1181, Biostatistics, Biomathematics, Pharmacoepidemiology and Infectious Diseases (B2PHI), Paris, France; ^2^Institut Pasteur, UMR 1181, B2PHI, Paris, France; ^3^Université de Versailles St Quentin, UMR 1181, B2PHI, Paris, France; ^4^Center of Cancer Systems Biology, Tufts University, Boston, MA, USA; ^5^Department of Environmental and Radiological Health Sciences, Colorado State University, Fort Collins, CO, USA; ^6^Department of Radiation Oncology, University of Texas Southwestern Medical Center, Dallas, TX, USA

**Keywords:** radiation, breast cancer cells, cancer stem cells, reprogramming, senescence, cellular automata

## Abstract

The enrichment of putative CD44^+^/CD24^−/low^ breast stem cell populations following exposure to ionizing radiation (IR) has been ascribed to their inherent radioresistance and an elevated frequency of symmetric division during repopulation. However, recent studies demonstrating radiation-induced phenotypic reprogramming (the transition of non-CD44^+^/CD24^−/low^ cells into the CD44^+^/CD24^−/low^ phenotype) as a potential mechanism of CD44^+^/CD24^−/low^ cell enrichment have raised the question of whether a higher survival and increased self-renewal of existing CD44^+^/CD24^−/low^ cells or induced reprogramming is an additional mode of enrichment. To investigate this question, we combined a cellular automata model with *in vitro* experimental data using both MCF-10A non-tumorigenic human mammary epithelial cells and MCF-7 breast cancer cells, with the goal of identifying the mechanistic basis of CD44^+^/CD24^−/low^ stem cell enrichment in the context of radiation-induced cellular senescence. Quantitative modeling revealed that incomplete phenotypic reprogramming of pre-senescent non-stem cells (reprogramming whereby the CD44^+^/CD24^−/low^ phenotype is conveyed, along with the short-term proliferation capacity of the original cell) could be an additional mode of enriching the CD44^+^/CD24^−/low^ subpopulation. Furthermore, stem cell enrichment in MCF-7 cells occurs both at lower doses and earlier time points, and has longer persistence, than that observed in MCF-10A cells, suggesting that phenotypic plasticity appears to be less regulated in breast cancer cells. Taken together, these results suggest that reprogramming of pre-senescent non-stem cells may play a significant role in both cancer and non-tumorigenic mammary epithelial populations following exposure to IR, a finding with important implications for both radiation therapy and radiation carcinogenesis.

## Introduction

Current dogma states that the recurrence of cancer in patients treated with radiation therapy is driven by the survival of radiation-resistant clonogens repopulating and replacing reproductively dead cells. This therapeutic resistance has been attributed to cells existing in hypoxic tumor regions where the lack of oxygen decreases the efficacy of radiation-induced cell killing. However, the contention that tumor-initiating or cancer stem cells, an inherently radioresistant population with increased DNA repair capacity, elevated expression of endogenous anti-oxidant defenses, and a slower rate of cell division are the potential drivers of this phenomenon, has prompted a good deal of interest in targeting cancer stem cells (1). Cancer stem cells were originally identified in acute myeloid leukemia (2, 3) and since have been identified in solid tumors and cell lines, including breast (4), prostate (5), lung (6), glioblastoma (7), and squamous cell carcinoma of the head and neck (8).

Previous studies have demonstrated that putative stem cells in normal and malignant mammary tissues are characterized by a CD44^+^/CD24^−^ phenotype (4, 9). Normal breast epithelial cells exhibiting the CD44^+^/CD24^−^ phenotype express genes associated with stem cells and somatic cell reprogramming at higher levels, and can asymmetrically divide and differentiate giving rise to sub-phenotypes of basal and luminal cells (10). Some human mammary epithelial cell lines, most notably MCF-10A non-malignant cells have been demonstrated the propensity to recapitulate ductal morphogenesis in the humanized fat pads of mice (11), offering strong evidence for a stem-like/progenitor subpopulation. *In vitro*, MCF-10A cells spontaneously acquire the CD44^+^/CD24^−^ phenotype via epithelial–mesenchymal transition (EMT) (12). In human breast cancers, the rare CD44^+^/CD24^−/low^ subpopulation shares properties with normal stem cells, including increased reproductive capacity and the ability to give rise to diverse cell lineages (4). CD44^+^/CD24^−^ cells isolated from some human breast cancer cell lines (e.g., MCF-7) and patient tumors demonstrate many stem-cell like properties *in vitro* and *in vivo* (13). Importantly, the purified CD44^+^/CD24^−^ cells (mesenchymal-like cancer stem cell state) are able to generate heterogeneous populations that recreate the proportion of CD44^+^/CD24^−^ and aldehyde dehydrogenase (ALDH) expressing cells (epithelial-like cancer stem cell state) present in the original cell lines (including MCF-7) (14), indicating that cellular plasticity enables breast cancer stem cells to transit between different phenotypes.

Radiation therapy is a common component of multimodal treatment designed to improve loco-regional control and overall survival in patients after breast-conserving surgery (15). After a single IR exposure (2–20 Gy γ-rays) we found the effective dose range for significantly enhancing the size of the stem cell pool differs between MCF-7 breast cancer cells and MCF-10A non-tumorigenic cells. Consistent with a previous report (16), following an acute radiation exposure of 10 Gy, the proportion of cells that are CD44^+^/CD24^−/low^ in both cell lines is elevated and peaks around day 5 after IR. This enrichment has been attributed to a higher radioresistance of CD44^+^/CD24^−/low^ cells and/or a switch from an asymmetric to symmetric type of division of CD44^+^/CD24^−/low^ cells, which then produce two identical CD44^+^/CD24^−/low^ daughter cells leading to a relative and absolute increase in CD44^+^/CD24^−/low^ subpopulation (17). In addition, Lagadec et al. demonstrated that radiation might reprogram a fraction of surviving non-stem committed cells (CCs) into the CD44^+^/CD24^−/low^ phenotype in some breast cancer cells (16). Notably, in our *in vitro* experiments, the fraction of senescent cells [cells that permanently withdraw from the cell cycle in response to diverse stress (18) (e.g., radiation-induced DNA damage), and can be identified by β-galactosidase (19)] increases and gradually dominates the population (~70%) during the 10 days post 10 Gy IR in both cell lines. The enrichment of stem cells in the irradiated populations prompted us to investigate how the fate of irradiated cells, in particular those experiencing IR-induced senescence, may influence cellular repopulation following exposure.

To explore the mechanistic basis for the elevated fraction of CD44^+^/CD24^−/low^ phenotype observed in normal and breast cancer cell populations following irradiation, we combined *in vitro* experiments with a cellular automata (CA) model to test mechanistic alternatives. Comparing simulation results with *in vitro* data demonstrated that neither (i) endowing normal and cancer stem cells with a lower radiosensitivity (i.e., a higher survival rate after irradiation), (ii) increasing the frequency of symmetric self-renewal division of stem cells, and (iii) increasing the rate of phenotypic reprogramming of surviving intact CCs to a full stem cell state, nor any combination of i, ii, and iii, were able to elevate the calculated stem cell percentage to match the observed percentage of CD44^+^/CD24^−/low^ cells following an acute dose of 10 Gy.

Unsuccessful model fitting based on the aforementioned hypotheses turned our attention to the potential contribution of IR-induced pre-senescent CCs (non-stem cells with short-term proliferation capacity due to radiation damage) to the replenishment of the stem cell pool through reprogramming. To this end, we considered two additional mechanisms: (iv) that, in addition to (iii), pre-senescent CCs can also be reprogramed to a stem cell state (i.e., CD44^+^/CD24^−/low^), albeit limited in this case to the remaining proliferative capacity they had before reprogramming (i.e., becoming pre-senescent SCs); or (v) all surviving CCs, whether pre-senescent, can have a potential to acquire a stem cell state with unlimited proliferative capacity. By fitting the model parameters in order to reproduce both the temporal dynamics of CD44^+^/CD24^−/low^ cells and the proportion of senescent cells in the population during the first 10 days after irradiation, we found that allowing for pre-senescent CCs to have additional reprogramming capability as described in mechanisms (iv) can explain experimental results not reconcilable with mechanisms (i)– (iii) or (v). Furthermore, we observed that IR induced a high reprogramming rate that lasted longer in MCF-7 cells compared to MCF-10A cells.

In conclusion, our study suggests that IR-induced incomplete phenotypic reprogramming of pre-senescent non-stem cells in irradiated MCF-10A and MCF-7 cells might be a contributing factor to the enrichment of the CD44^+^/CD24^−/low^ phenotype. Incomplete phenotypic reprogramming of pre-senescent CCs also gives rise to a heterogeneous stem cell pool consisting of a fraction of cells that express the stem cell marker, but have a short-term proliferative potential. Finally, we find MCF-7 breast cancer cells to be more sensitive to acute, high-dose IR than MCF-10A non-tumorigenic mammary epithelial cells in terms of phenotypic reprogramming.

## Materials and Methods

### Cell Culture

The human mammary epithelial, non-tumorigenic cell line MCF-10A was purchased from ATCC and cultured in 1:1 Dulbecco’s modified essential medium (DMEM)/Ham’s F12 growth medium (Hyclone) supplemented with 5% fetal bovine serum (FBS), 10 μg/mL insulin (Sigma), 20 ng/mL epidermal growth factor (EGF;Sigma), 0.5 μg/mL hydrocortisone (Sigma), 0.1 μg/mL cholera toxin (Sigma), and 1% glutamax (Life Technologies). The human mammary carcinoma cell line MCF-7 (kind gift from L. Chubb, CSU Animal Cancer Center) was grown in DMEM supplemented with 10% FBS and 1% glutamax (Life Technologies). Cells were grown at 37°C in a humidified incubator at 5% CO_2_ passaged 1–2 times per week.

### Mammosphere Assay

Sphere-forming assay was utilized to confirm stem-like properties of MCF-7 cells. Briefly, monolayer cultures were grown in low adherence dishes (Corning) at a low density in Mammocult sphere-forming media (Stem Cell Technologies). Limiting dilution assays were performed in 96-well plates comparing sorted CD44^+^/CD24^−/low^ to bulk monolayer cell cultures. Spheres were allowed to form for 10 days, and spheres larger than 60 μm in diameter were scored (20).

### Irradiations

For clonogenic cell survival assays, cells were seeded 48 h prior to irradiation at a density of 3 × 10^5^ cells per T25 flask. Monolayer cultures were irradiated in a Mark I Irradiator (J.L. Shepherd) utilizing a Cs-137 source at acute doses of 1, 2, 4, 5, 8, and 10 Gy or sham irradiated as a control. Following irradiation, cells were allowed to repair for 6 h and plated in triplicate at low density in 100-mm cell culture plates (Greiner) containing 10 mL of culture medium. Cells were incubated for 12 days (MCF-7) or 16 days (MCF-10A), fixed in 100% ethanol, and stained with 0.05% crystal violet solution. Colonies were scored based on the presence of 50 or more cells and scored independently by two individuals.

### Flow Cytometry Analysis

All flow cytometry analyses were performed at the Colorado State University Animal Cancer Center on a Beckman Coulter CyAN ADP 9 Color analyzer running Summit Version 3.0 flow cytometry analysis software. Mammary epithelial stem cells were identified based on expression of CD44^+^/CD24^−/low^ immunotype and stem-like properties were confirmed utilizing the ALDEfluro Assay (Stem Cell Technologies). Monolayer MCF-7 and MCF-10A mammary epithelial cell cultures were stained for CD44 and CD24 expression. Briefly, ~3 × 10^5^ cells were dissociated from cell culture surface using 0.25% Trypsin-EDTA, pelleted, washed, and re-suspended in 30 μL of Flow Cytometry wash buffer (1X PBS, 1% FBS, and 1% Penicillin/Streptomyocin). Six microliters of direct FITC-conjugated mouse monoclonal anti-human CD44 antibody (BD Pharmingen #555478) and 6 μL of direct PE-conjugated mouse monoclonal anti-human CD24 antibody (BD Pharmingen #555428). Cells were then incubated for 30–60 min in the dark at 4°C. Following incubation, cells were pelleted and re-suspended in 500 μl of cold 1 × PBS and kept on ice until analysis. Analysis gates were established using cells from an unstained control and anti-mouse Ig,κ antibody capture beads (BD Pharmingen #552843).

### Identification of Senescent Cell Fraction

The fraction of senescent cells was determined via β-galactosidase staining. Cells were stained using β-galactosidase staining kit (Cell Signaling Technologies) and imaged on a confocal microscope at a 20× magnification utilizing a color camera. Cells were scored as positive based on the presence of blue pigmentation in the nuclei of adherent cells.

### Cellular Automata Model

A CA model is used to simulate the dynamics and interactions of single cells in the growth of cell population (21). In a CA model, a system is represented as a collection of autonomous decision-making agents (e.g., cells). Each agent is endowed some intrinsic state variables and behaves and interacts with each other and its external environment given a set of predefined rules. Stochastic interactions of single cells as well as with their external environment result in complex population dynamics. The CA framework can capture the interactive consequences of these dynamics while allowing for the examination of phenotypical and functional heterogeneity, such as stem cell biology.

The system is defined on a two-dimensional square lattice (*L* × *L* lattice points) with periodic boundary conditions. As *in silico* cells live on a square lattice with (15 μm)^2^ grid points, a single migration step to a neighboring location is calibrated as 15 or 21 μm in an 8-cell Moore neighborhood (21). Each lattice point can stay empty or be occupied by one cell. If a free lattice site is found within the Moore neighborhood of a cell, it can migrate with a probability *p*_m_, or divide to produce a new cell provided the maturation has been reached. A proliferative cell turns quiescent when it is completely surrounded by other cells but can re-enter the cell cycle when a neighboring free space is available. As per the stem cell hypothesis, stem cells reside at the top of the hierarchy and produce progenitor cells, which in turn give rise to CCs. For present purposes, a CC will refer to a non-stem cell. A stem cell is capable of dividing into two stem cells (symmetric division) with probability *p*_S_, or a stem cell and a CC with probability 1 − *p*_S_ (asymmetrical division). The duplication of a non-senescent CC results in two CCs. A CC ceases to divide after some number of divisions, a phenomenon known as replicative senescence, or the Hayflick limit. The parameter ρ will refer to the number of remaining divisions a cell is capable of undergoing before it becomes senescent (the value ρ for a stem cell is, thus, ρ = ∞).

It has been found that normal and neoplastic non-stem cells can spontaneously convert to a stem-like state (22). In our model, CCs are eligible for reprogramming with probability *p*_r_. The rate of symmetric division (*p*_S_) and reprogramming rate (*p*_r_) were estimated together in order to match the control frequency of CD44^+^/CD24^−/low^ in both cell lines (Figure S1 in Supplementary Material). We referred a stem cell study by Tang et al. (11) in order to guess the initial value of *p*_S_.

To reproduce the population dynamics *in vitro*, we began with a fixed division cycling time in the CA model. However, the resultant growth curves were exponential, which could not explain the population dynamics *in vitro*, in which the cell proliferation rate decreases at later time points (Figure S2 in Supplementary Material). Indeed, cell population growth can be affected by the surrounding environment, such as (a) cell–cell contact inhibition (already implemented in the CA model), (b) nutrient availability, and (c) accumulation of toxic wastes. In our experiments, fresh culture medium was added to the cultures to ensure adequate cellular nutrition levels. However, no cell culture medium was removed following irradiation in order to prevent removal of potential reprogramming signals. Therefore, it is reasonable to presume that the decreasing proliferation rate of control growth (sham irradiated) is mostly a result of *c*. To depict this phenomenon simply, we introduced a cell cycle regulation mechanism implicitly as a function of cell population density:

$$\text{Cell cycle = }c_{0}e^{k\text{*(population density)}}$$

where *c*_0_ is the initial cycling time and *k* is the inhibition coefficient. The values of *c*_0_ and *k* were estimated by matching the population dynamics *in vitro* (Figure S3 in Supplementary Material). The estimated *k* value was smaller for MCF-7 cells than MCF-10A cells (Table 1) suggesting that breast cancer cells are more resistant to a stressful microenvironment than non-cancerous cells, which belongs to one hallmark of cancer (evading growth suppressors) (23).

**Table 1:** **Model parameters and values**.

Parameter	Meaning	Value		Reference
		MCF-10A	MCF-7	
*R*	MCF-10A or MCF-7 cell diameter	15 μm	15 μm	(24)
*V*	Cell migration speed	23.4 μm h^−1^	23.4 μm h^−1^	(25, 26)
*c_0_*	Initial cell cycle	17 h	24 h	Fitting *in vitro* data
*k*	Cell cycle inhibition coefficient	18	2.97	Fitting *in vitro* data
*p*_d_	Spontaneous cell death rate of CC	0.004 CC^−1^day^−1^	0.02 CC^−1^day^−1^	Fitting *in vitro* data
*p*_s_	Symmetric self-renewing division rate	10% SC (C)	10% (C)	Fitting *in vitro* data
*p*_r_	Phenotypic reprogramming rate	0.004 CC^−1^day^−1^ (C)	0.0017 CC^−1^day^−1^(C)	Fitting *in vitro* data
*p*_a_	Probability of permanent arrest	0.63	0.76	Fitting *in vitro* data
*SF*	Overall survival rate following acute 10 Gy IR	0.0071	0.0031	Clonogenic assay
ρ_psn_	Proliferation potential of pre-senescent cells	2–6 divisions	0–9 divisions	Fitting *in vitro* data

*C, control/sham irradiated with 0 Gy*.

**Figure 2:**
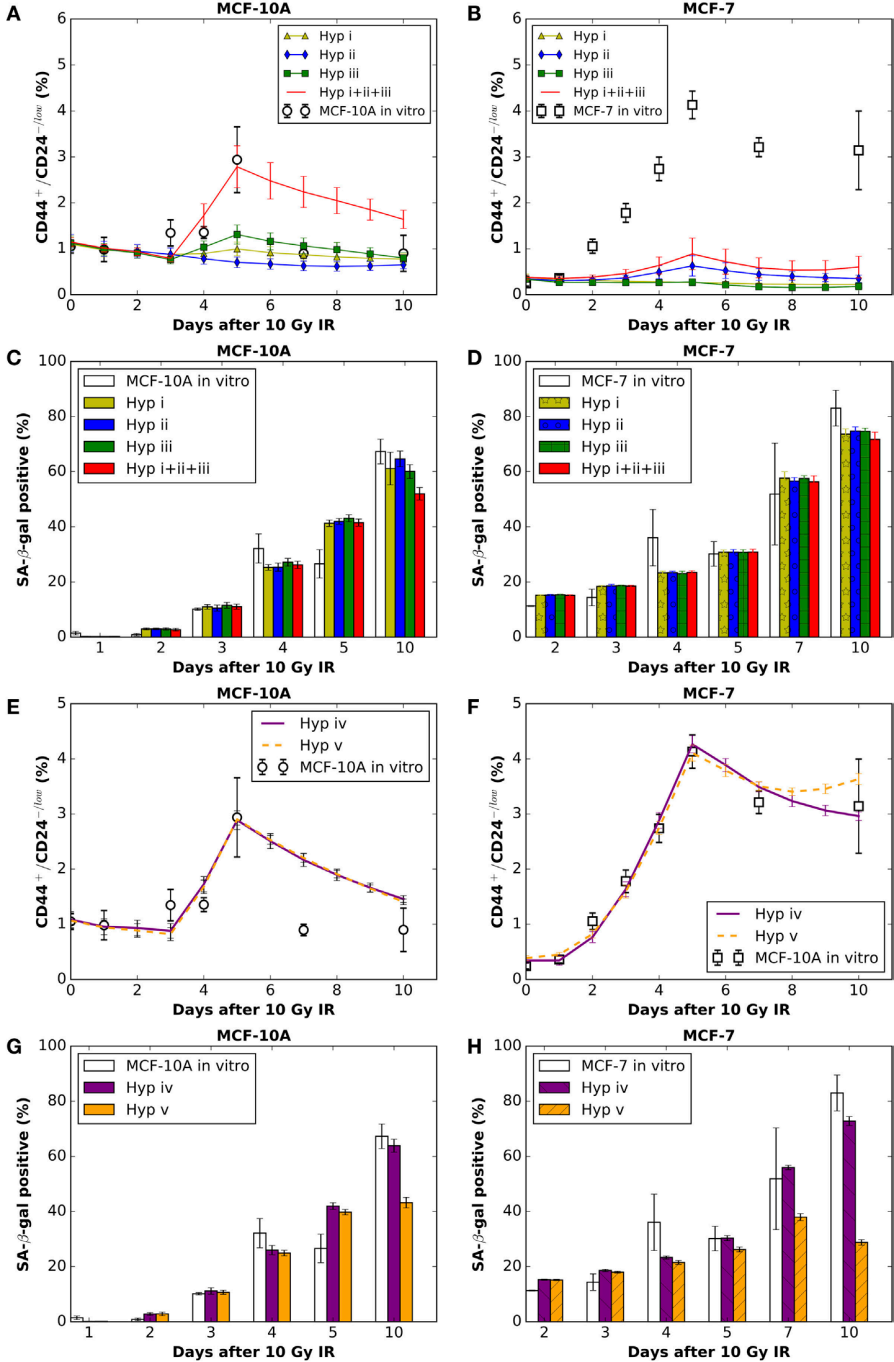
**The comparisons between model simulation results (mean ± SD; *n* = 10 simulations) of applying hypothesis (i), (ii), or (iii) (alone or in combinations) and *in vitro* data (mean ± SD; *n* = 3) on the (A)% of CD44^+^/CD24^−/low^ cells in MCF-10A cells and (B) MCF-7 cells; and the (C)% of SA-β-gal positive cells in MCF-10A cells and (D) MCF-7 cells**. The comparisons between simulation results of applying hypothesis (iv) or (v) and *in vitro* data on the **(E)**% of CD44^+^/CD24^**−**/low^ cells in MCF-10A cells and **(F)** MCF-7 cells; and the **(G)**% of SA-β-gal positive cells in MCF-10A cells and **(H)** MCF-7 cells. Hyp stands for hypothesis in the figure legends. Best fitting for MCF-10A cells under Hyp (iv) [corresponding to **(E,G)**]: reprogramming rate (*p*_r_) increases to 0.09 per CC (intact or pre-senescent) per day for 38 h during days 3–5 after irradiation. Best fitting for MCF-7 cells under Hyp (iv) [corresponding to **(F,H)**]: reprogramming rate (*p*_r_) increases to 0.08 per CC (intact or pre-senescent) per day during first 4 days after irradiation, then decreases to 0.0198 for the following time points.

After radiation exposure, cells exhibit mitotic delay while attempting to repair radiation-induced DNA damage (27). Several studies have demonstrated that a large fraction of normal fibroblasts irradiated in G1-phase and reseeded after irradiation, do not re-enter the cell cycle but remained permanently arrested (28–30). Accordingly, we assumed that a cell either undergoes a permanent cycle arrest with a probability *p*_a_ or experiences transient arrest for a randomly chosen time between 0 and 10 days following radiation exposure. Cell survival probability after irradiation was determined via clonogenic assay (Figure S4 in Supplementary Material). Some studies have demonstrated that MCF-7 cells undergo mostly IR-induced senescence instead of apoptosis (31–33). By day 10 of our experiment following a 10 Gy single-dose IR, the senescent phenotype (SA-β-gal positive cells) had increased not only in ratio but also in number relative to day 0 within the proliferating cell population for both cell lines (Figures 2C,D; Figure S3 in Supplementary Material), which indicates the existence of cells in a pre-senescent state (have short-term proliferations before undergoing a senescent state). Therefore, we assigned to the pre-senescent CCs and pre-senescent SCs a temporal range of short-term proliferation potential ρ_psn_. The range of values for ρ_psn_ was estimated by matching the irradiated population dynamics (Figure S3 in Supplementary Material) and senescent cell fractions *in vitro* (Figures 2C,D). Cell cycle arrest has been found to prevent reprogramming (34, 35). Hence, a reprogramming rate *p*_r_ = 0 was applied to CCs in an arrested state and senescent cells.

A diagram of the simulation process and decisions at the cell level is shown in Figure S5 in Supplementary Material. The model parameters and their values are summarized in Table 1.

## Results

### MCF-10A and MCF-7 Populations Show Different Dose Ranges Over Which There is Substantial CD44^+^/CD24^−/low^ Cell Fraction Modification

**Figure 1:**
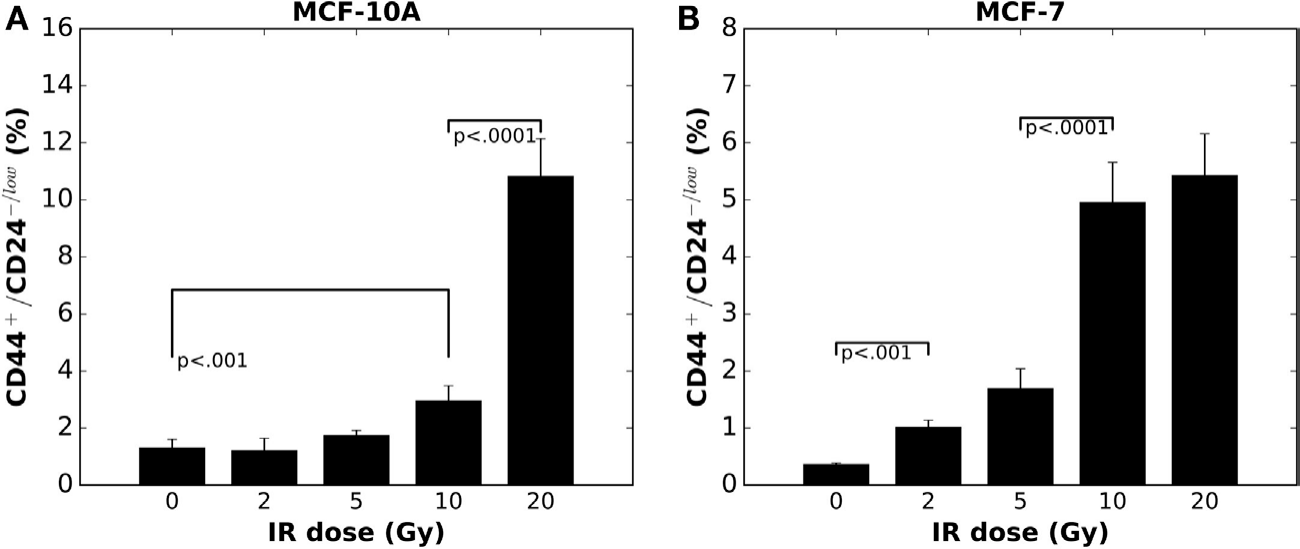
**Radiation-induced enrichment of CD44^+^/CD24^−/low^ putative stem cells in (A) MCF-10A cells and (B) MCF-7 cells *in vitro* is in a dose dependent manner (mean ± SD; *n* = 3)**.

In MCF-10A cells, at day 5 after irradiation with a single dose of 5 Gy or lower, the fraction of CD44^+^/CD24^−/low^ cells showed no difference compared to control (Figure 1A); whereas a dose as low as 2 Gy induced an enrichment of the CD44^+^/CD24^−/low^ subpopulation among MCF-7 cells (Figure 1B) (20). When the IR dose increased to 20 Gy, a tremendously high enrichment of CD44^+^/CD24^−/low^ subpopulation appeared in MCF-10A cells (Figure 1A). By contrast, a 20 Gy single dose did not further increase the fraction of CD44^+^/CD24^−/low^ subpopulation in MCF-7 cells (Figure 1B). Presumably, the mechanisms for regulating the CD44^+^/CD24^−/low^ cells are not easily altered in the non-tumorigenic population vs. cancer cells, suggesting a potentially pivotal role for this enrichment in tumor re-growth following radiation therapy.

### Enrichment of CD44^+^/CD24^−/low^ Phenotype in MCF-10A and MCF-7 Cells May Not Derive Purely from Intact Surviving Cells

To evaluate the mechanistic basis of IR-induced enrichment of CD44^+^/CD24^−/low^ phenotype in MCF-10A and MCF-7 cells, we tested the following hypothesis: (i) low radiosensitivity of stem cells (maximum tested: 100% survival after radiation exposure), (ii) increased symmetric division frequency (maximum tested: 100% per stem cell division) of stem cells, and (iii) increased reprogramming frequency of undamaged cycling CCs (maximum tested: 100% per intact CC per day). The resulting fractions of senescent cells were similar to one another for the three mechanisms, which roughly reproduced the observed dynamics of state with a decreased reproductive capacity positive cell staining *in vitro* (Figures 2C,D). Surprisingly, however, neither of the three mechanisms alone were able to produce the observed high percentage of CD44^+^/CD24^−/low^ cells observed in either cell line (Figures 2A,B), nor were combinations of any two mechanisms able to generate the high fraction of stem cells especially at day 5 after exposure to 10 Gy (data not shown). Combining all three mechanisms (e.g., >50% survival rate of SCs, 100% symmetric division rate, and 100% reprogramming rate of non-arrested intact CCs) produced a comparable fraction of CD44^+^/CD24^−/low^ in MCF-10A cells (Figure 2A) but not in MCF-7 cells. However, the resulting ratio of senescent cells (~43% *in silico*) was smaller than that observed (67% *in vitro*; Figure 2C) at day 10 after irradiation, which makes it less likely that the possibility of the combination of above three mechanisms is a major force in enriching the stem cell pool.

### Radiation-Induced Incomplete Phenotypic Reprogramming of Pre-Senescent Non-Stem Cells Appears More Likely To Be an Additional Mode of Enriching CD44^+^/CD24^−/low^ Cells

**Figure 3:**
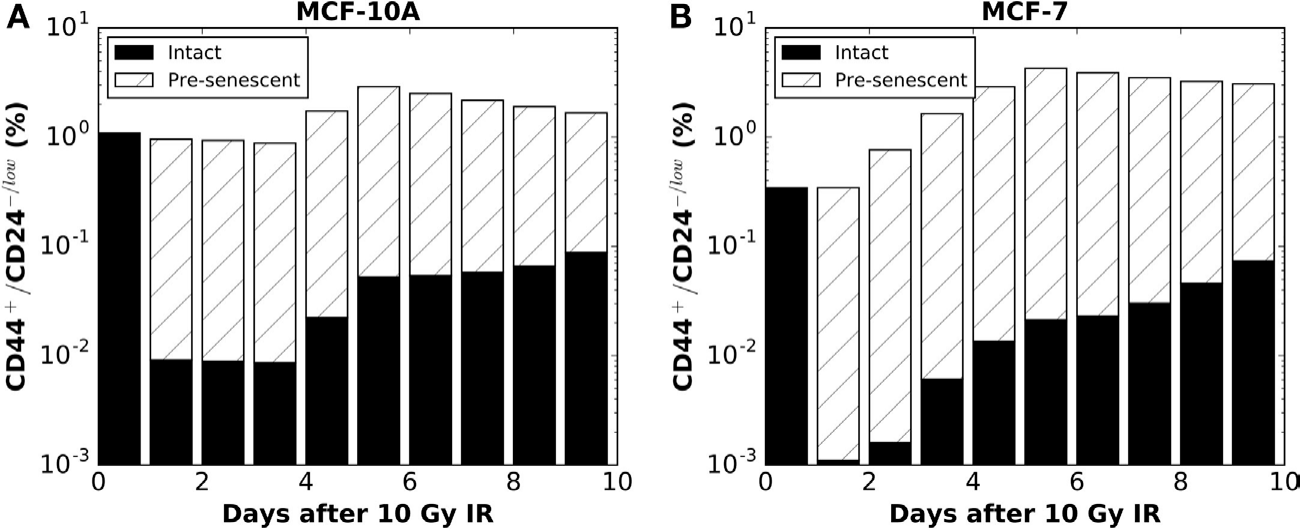
**Following the hypothesis of IR-induced incomplete phenotypic reprogramming, the model simulation predicted the proportions of intact (solid colors) vs. pre-senescent (striped colors) CD44^+^/CD24−/low sub-populations for **(A)** MCF-10A cells (corresponding to Hyp (iv) curve in Figure 2E) and **(B)** MCF-7 cells [corresponding to Hyp (iv) curve in Figure 2F]**. Data points are plotted on a log scale.

The above disparities led us to consider alternative explanations; specifically, phenotypic reprogramming of pre-senescent non-stem cells. In the latter case, it has been demonstrated that cellular senescence is not a limit to reprogramming and that age-related cellular physiology is reversible (36). Nevertheless, it is unknown to what extent reproductive potential can be regained by the reprogramming of senescent cells, e.g., 0–100%. Instead of testing the recovery of proliferative capacity in a quantitative manner, we simply assume that reprogramming of a pre-senescent CC can be either (iv) incomplete (i.e., pre-senescent CCs are reprogramed to pre-senescent SCs but inherit the remaining proliferative potential) or (v) complete (i.e., pre-senescent CCs are reprogramed to SCs and reacquire unlimited proliferative potential). Simulation results showed that both hypotheses (iv) and (v) could successfully reproduce a comparable ratio of CD44^+^/CD24^−/low^ cells observed 10 days after 10 Gy (Figures 2E,F). However, following hypothesis (v), at day 10 after 10 Gy, the fraction of senescent cells in simulations was lower than observed *in vitro* in both cell lines (Figures 2G,H). Considering these findings together, the enrichment of CD44^+^/CD24^−/low^ phenotype in MCF-10A and MCF-7 cells is more likely driven to a large extent by incomplete phenotypic reprogramming. As a consequence, the enriched stem cell pool would be mixed with a fraction of stem cells that have a short-term proliferative potential (Figure 3). Therefore, the signature of CD44^+^/CD24^−/low^ is no longer accurate for presenting the overall “stemness” of the irradiated cell population.

### The IR-Induced Reprogramming Events Persist Longer in MCF-7 Breast Cancer Cells than in MCF-10A Non-Tumorigenic Mammary Epithelial Cells

During the process of parameter fitting in order to reproduce the dynamics of the CD44^+^/CD24^−/low^ subpopulation after a 10 Gy single-dose IR, we found differential changes in the kinetics of reprogramming between MCF-10A and MCF-7 cells. For MCF-10A cells, the fitted reprogramming rate only transiently increased to 0.09 per CC (intact or pre-senescent) per day for 38 h during days 3–5 after IR (Figure 2E). By contrast, the reprogramming rate of MCF-7 cells increased to 0.08 per CC (intact or pre-senescent) per day immediately following the radiation exposure and through day 4 (Figure 2F), then decreased to 0.0198 per CC (intact or pre-senescent) per day although it remained elevated relative to the control (0.0017 per CC per day; Table 1).

Taken together, these results demonstrate that the equilibrium of CD44^+^/CD24^−/low^ cells is more tightly regulated in non-tumorigenic than cancer cells in response to an acute 10 Gy dose of radiation; deregulation of this process may play a role in carcinogenesis by providing an advantage to cells that are more capable of being reprogramed to a stem-like state.

## Discussion and Conclusion

Maintenance of the pool of putative stem cells requires a finely tuned balance between self-renewal, differentiation, and recruitment (dedifferentiation or reprogramming). Alterations in the equilibrium of maintaining adult stem cells can affect tissue homeostasis as well as cancer progression and carcinogenesis. Radiation-induced enrichment of stem cells has been attributed to advanced DNA-damage repair mechanisms (37, 38), enhanced survival and subsequent expansion of the (more resistant) quiescent fraction of stem cells as they return to a proliferative state (39), a switch from asymmetric to symmetric stem cell self-renewal division (40), and an increased frequency of reprogramming (16, 41). For this study of MCF-10A and MCF-7 cells, a CA model was used to test several hypotheses, including (i) lower radiosensitivity (or higher survival rate) of SCs, (ii) increased symmetric division frequency, (iii) increased phenotypic reprogramming frequency of intact non-arrested CCs, (iv) incomplete reprogramming of pre-senescent CCs to pre-senescent SCs with short-term proliferative capacity, and (v) complete reprogramming of pre-senescent CCs to SCs with unlimited proliferative capacity. Our simulation results showed that incomplete phenotypic reprogramming (hypothesis iv) could reproduce the dynamics of CD44^+^/CD24^−/low^ cells, as well as the fraction of SA-β-gal positive senescent cells *in vitro* for both cell lines (20). Following IR-induced incomplete phenotypic reprogramming, the resultant stem cell pool is expected to be heterogeneous, with the reprogramed cells expressing putative stem cell markers (CD44^+^/CD24^−/low^), but possessing only short-term proliferation potential. Therefore, such heterogeneity would suggest that a large stem cell pool may not necessarily implicate a strong population re-growth potential if a high proportion of stem cells have a short-term proliferation potential. To test this hypothesis, we plan to purify the IR-enriched stem cells, and then compare their colonization and mammosphere formation capacity with unirradiated stem cells. It has been previously demonstrated that in primary breast xenografts, CD44^+^/CD24^−^ and ALDH expressing cells identified overlapping, but non-identical cell populations, both of which were able to initiate tumors in NOD/SCID mice (42). Importantly, ALDH^+^ and CD44^+^/CD24^−/low^ cells can transit between each other via EMT and mesenchymal–epithelial transition (MET) (14), highlighting the necessity of using both CD44/CD24 and ALDH for measuring changes in stem cell pool size after irradiation. Additionally, co-staining with β-gallactosidase will determine whether senescent cells express stem cell markers.

In our previous study (40), we showed that radiation-induced accelerated proliferation of glioma stem cells may contribute to their increased frequency in recurrent glioblastoma. To our knowledge, IR-induced proliferation of CD44^+^/CD24^−/low^ compartments *in vitro* lacks experimental support. Indeed, Wicha and colleagues (14) have shown that the CD44^+^/CD24^−^ signature is associated with a low proliferative capacity. However, if we assume expansion of CD44^+^/CD24^−/low^ cellular compartments via accelerated symmetric division after radiation exposure, some would be expected to localize adjacently, a prediction that could be confirmed by co-localization of CD44^+^/CD24^−/low^ cells after irradiation.

Phenotypic plasticity appears to be more tightly regulated in MCF-10A non-tumorigenic mammary epithelial cells in response to an acute radiation exposure of 10 Gy or lower. In contrast, relatively high plasticity can be induced in MCF-7 breast cancer cells by a lower dose if IR (i.e., ≤10 Gy). When dose is increased to 20 Gy, an elevated level of cellular reprogramming might be evoked in the normal breast epithelium cells, resulting in enrichment of stem cell pools. The reprogramming capacity of breast cancer cells seems to reach a plateau at 10 Gy, beyond which no significant increase in the percentage of CD44^+^/CD24^−/low^ cells is observed. Notably, the IR-induced high rate of phenotypic reprogramming lasted longer in MCF-7 cells than MCF-10A cells, where it appears only transiently.

Molecular mechanisms governing reprogramming in the context of radiation therapy remain elusive, although our recent study demonstrated that IR-induced stem cell enrichment is telomerase dependent (20). Genomic analysis of cell populations at the time points corresponding to the modeling done here may strengthen the case for IR-induced reprogramming. Specifically, the difference in reprogramming response with regard to non-cancer vs. cancer cells could reflect deregulation of anti-tumor molecular machinery such as occurs during the process of carcinogenesis, which may also tightly regulate the ability of cells to be reprogramed, providing fertile ground to explore new mechanisms driving this disease. Wicha and colleagues suggested HER2 as a potential driver of cancer stem cells in luminal breast cancers (43). They showed that knockdown of HER2 abrogates MCF-7 cells tumorsphere formation. According to a study by Chung et al. (44), HER2 can induce stem cell marker expression and SLUG upregulation that promote the EMT phenotype in MCF-7 cells. Indeed, HER2 is overexpressed in MCF-7 cells following IR (45). Quantitative measurement of HER2 expression levels and kinetics in senescent normal epithelial and breast cancer cells may provide invaluable information on the role of senescent cells in regulating cellular population dynamics after ionizing radiation exposure.

## Ethics Statement

No animal or human subjects were utilized in this research. All other use of human materials (ie. cell lines) was conducted according to standard biosafety and ethics guidelines at Colorado State University.

## Author Contributions

Design of the work: XG, BS, SB, PH, and LH. Drafting the work: XG and BS. Data acquisition: BS and CN. Data analysis: XG and BS. Revising the work critically for important intellectual content: PH, LH, and SB. Final approval of the version to be published: PH, SB, and LH.

## Conflict of Interest Statement

The authors declare that the research was conducted in the absence of any commercial or financial relationships that could be construed as a potential conflict of interest.

## Supplementary Material

Figure S1 **Simulation reproducing the fraction of CD44^+^/CD24^-/low^ cells in the control 494 condition (sham irradiation) for (A) MCF-10A cells and (B) MCF-7 cells (mean ± SD; *n* = 10 simulations)**.

Figure S2 **Unsuccessful fitting of cell population dynamics by applying reported 496 average cell cycle time *in vitro* for both (A) MCF-10A cells (fitting curve: average cell cycle time 20 497 hours (46); mean ± SD; *n* = 10 simulations) and (B) MCF-7 cells (fitting curve: average cell cycle time 498 26.8 hours (47); mean ± SD; *n* = 10 simulations)**.

Figure S3 **Simulation reproducing population dynamics with sham irradiation or a 10 500 Gy single-dose IR for (A) MCF-10A cells and (B) MCF-7 cells (mean ± SD; ***n*** =10 simulations)**. Hyp 501 stands for hypothesis in the figure legends.

Figure S4 **Clonogenic survival fraction of (A) MCF-10A cells and (B) MCF-7 cells 503 and fitted curve with linear quadratic equation**.

Figure S5 **Diagram of the simulation process and decisions on the cell level**.
