# Supplementary figures and images for: Radiation-Induced Reprogramming of Pre-Senescent Mammary Epithelial Cells Enriches Putative CD44+/CD24−/low Stem Cell Phenotype
Source: Front Oncol. 2016 Jun 14;6:138. doi: 10.3389/fonc.2016.00138 (PMC4905979; doi:10.3389/fonc.2016.00138)

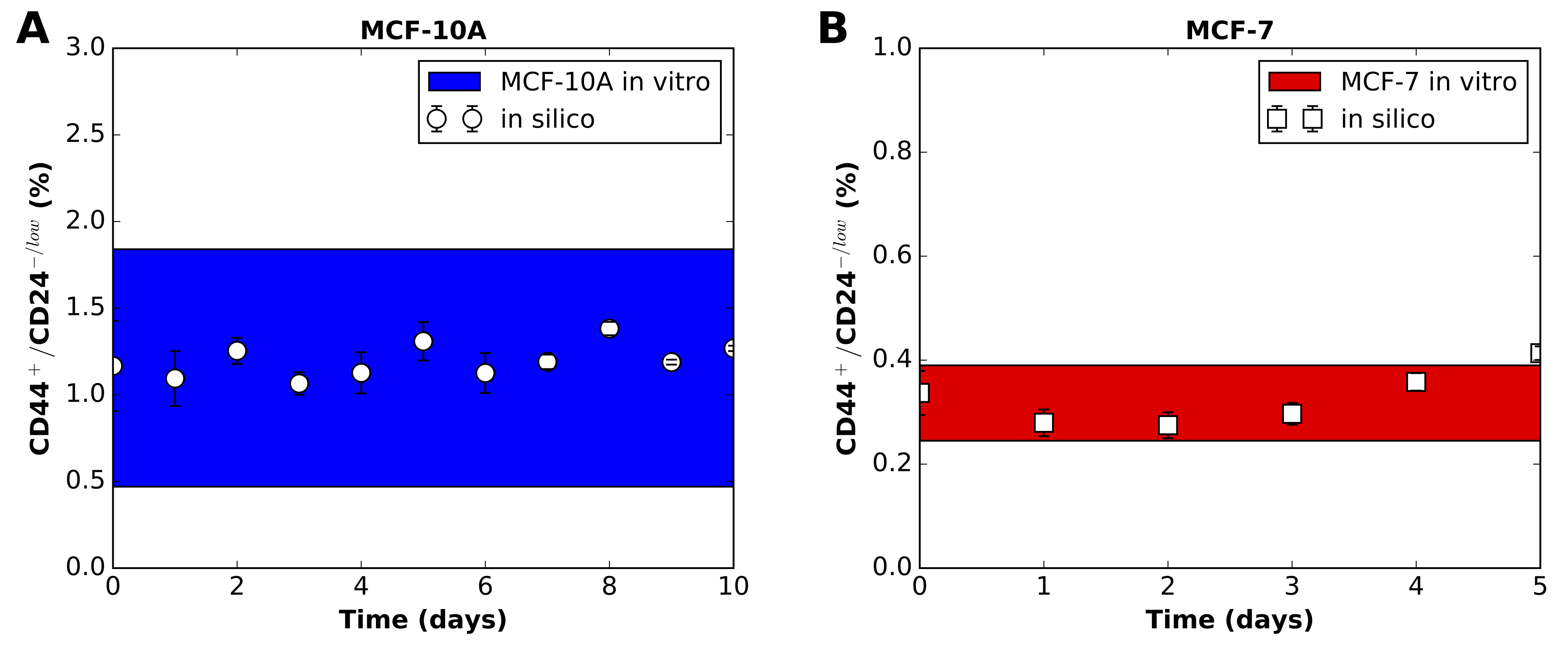

Supplement: Figure S1 — Simulation reproducing the fraction of CD44+/CD24-/low cells in the control 494 condition (sham irradiation) for (A) MCF-10A cells and (B) MCF-7 cells (mean ± SD; n = 10 simulations). [file image_1.jpeg]

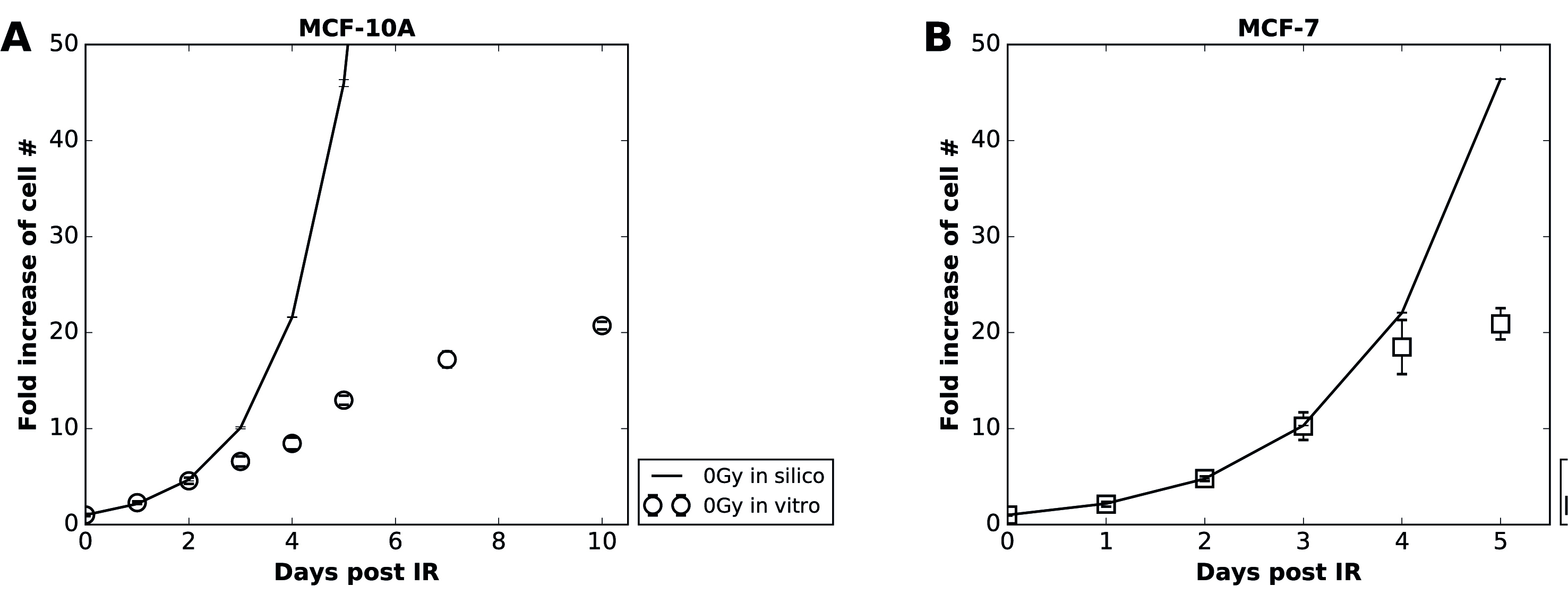

Supplement: Figure S2 — Unsuccessful fitting of cell population dynamics by applying reported 496 average cell cycle time in vitro for both (A) MCF-10A cells (fitting curve: average cell cycle time 20 497 hours (46); mean ± SD; n = 10 simulations) and (B) MCF-7 cells (fitting curve: average cell cycle time 498 26.8 hours (47); mean ± SD; n = 10 simulations). [file image_2.jpg]

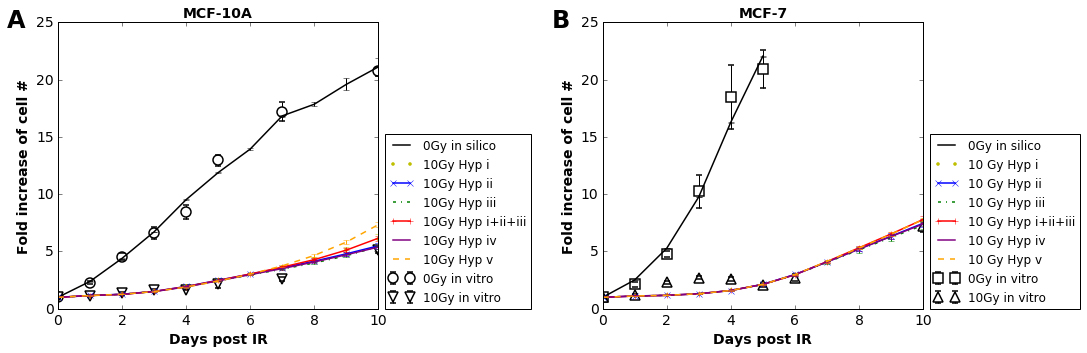

Supplement: Figure S3 — Simulation reproducing population dynamics with sham irradiation or a 10 500 Gy single-dose IR for (A) MCF-10A cells and (B) MCF-7 cells (mean ± SD; n =10 simulations). Hyp 501 stands for hypothesis in the figure legends. [file image_3.jpg]

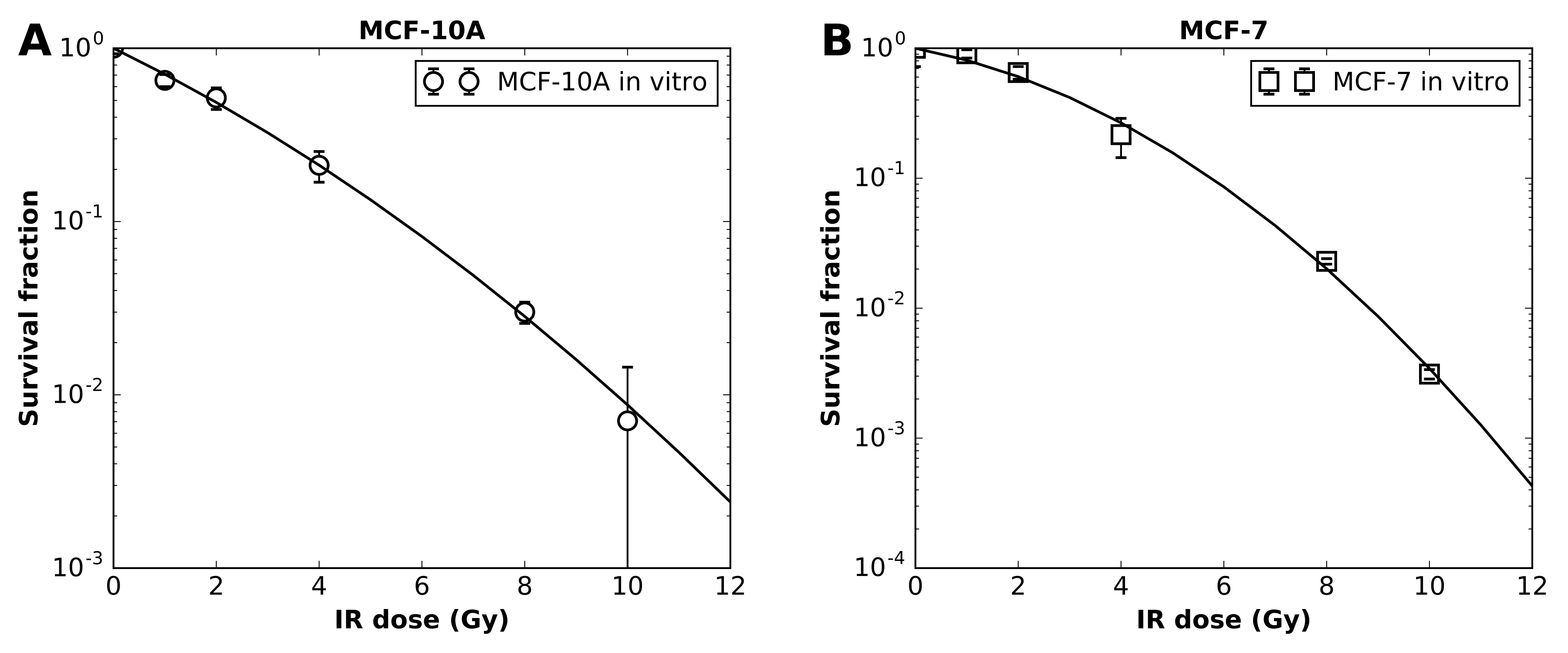

Supplement: Figure S4 — Clonogenic survival fraction of (A) MCF-10A cells and (B) MCF-7 cells 503 and fitted curve with linear quadratic equation. [file image_4.jpeg]

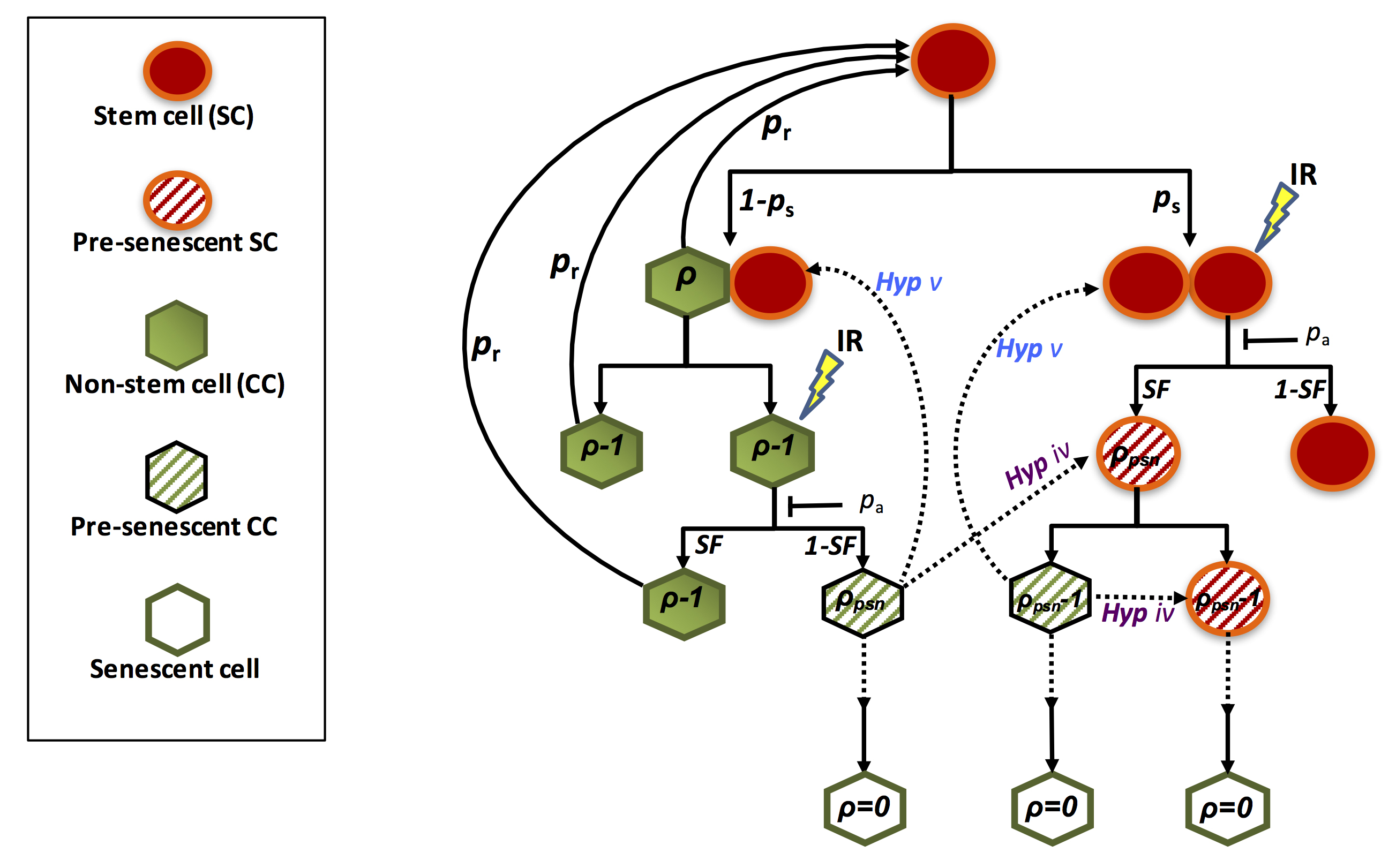

Supplement: Figure S5 — Diagram of the simulation process and decisions on the cell level. [file image_5.jpg]
